# Supplementary material for: Effect of Vitamin D Supplementation on the Fetal Growth Rate in Pregnancy Complicated by Fetal Growth Restriction
Source: Children (Basel). 2022 Apr 12;9(4):549. doi: 10.3390/children9040549 (PMC9025187; doi:10.3390/children9040549)
Supplement: Supplementary file 1 [file children-09-00549-s001.zip › children-1634426-supplementary.pdf]

SURVEY FOR THE RESEARCH PARTICIPANT

"The effect of vitamin D supplementation on the growth rate of the fetus with growth restriction"

You have been qualified and agreed to participate in the study "The effect of vitamin D supplementation on the growth rate of a fetus with growth restriction", therefore please complete a short questionnaire to determine the amount of vitamin D you are taking. I kindly ask you to provide your data and answer the following questions.

First name and last name:………………………………………………………………………………...

Date of birth:………………………………………………………………………………..

A) What vitamin supplements do you currently use:

1. Pregna Plus []

2. Mother DHA []

3. Prenatal Clasic / DUO []

4. Falvit Mama []

5. Femibion []

6. Vita-min plus mama []

7. Doppelherz activ mama []

8. Other [], please specify the name ……………………………… ..

9. I do not use []

B) How do you dose vitamin supplements, i.e. how many times a day? ………………………..

C) From which week of pregnancy do you take vitamin supplements? ....

D) Do you take them regularly?

1.yes, everyday []

2.yes, every second day []

3.yes, but sometimes I forget to take a single dose [], how often? ……………

3.no [], please describe how ……………………………………………………

E) Do you also take vitamin D alone?

1. No []

2. Yes [], please provide the name of the preparation ………………………………………………

F) How do you dose vitamin D, i.e. how many times a day? ………………………………………….

G) From which week of pregnancy do you take vitamin D? ....

H) Do you take it regularly?

1.yes, everyday []

2.yes, every second day []

3.yes, but sometimes I forget to take a single dose [], how often? ……………

3.no [], please describe how ………………………………………………… ..… ..
